# Intralesional combined digoxin and furosemide versus intralesional 5-flurouracil for the treatment of recalcitrant plantar warts: a prospective, randomized study

**DOI:** 10.1007/s00403-024-03014-z

**Published:** 2024-06-15

**Authors:** Fathia khattab, Reham Essam, Reham F. Elhadidy, Nourhan Anis

**Affiliations:** Dermatology, Venereology and Andrology Department, Faculty of Medicine, Zagazig University, Zagazig, Egypt

**Keywords:** Combined digoxin and furosemide, Dermoscopy, 5-fluorouarcil, Plantar warts

## Abstract

There are many therapeutic modalities for plantar warts, however treating it remains challenging. Intralesional injection of 5-fluorouarcil and combined digoxin and furosemide were observed to be effective and safe, however no comparison study between them was done. Our study was conducted to evaluate the efficacy of both therapies in the treatment of plantar warts. 90 adult patients with multiple recalcitrant plantar warts were included in our study. They were randomly allocated to one of three groups; combined digoxin and furosemide, 5-fluorouarcil, or normal saline group. Fortnightly injections were done into all studied warts till complete clearance or up to 5 sessions. Warts were evaluated clinically and dermoscopically. Clinical response was reported in 24 patients (80%) of the combined digoxin and furosemide group with 40% complete response and in 24 patients (80%) of the 5-fluorouarcil group with 33.3% complete response. No statistically significant difference was observed between the two groups concerning efficacy and safety. Intralesional injection of 5-fluorouarcil and combined digoxin and furosemide are nearly equivalent in efficacy and safety for plantar wart treatment. Dermoscopy helps to take the truthful judgment about complete clearance of warts.

## Introduction

Warts are benign growth of skin and mucosa produced by the human papilloma virus (HPV). Plantar warts are one of the main clinical types of cutaneous warts [1]. The primary motives to pursue therapy for plantar warts include pain, function impairment, transmission risk, and cosmetic embarrassment [2]. There are many therapeutic modalities for warts mentioned in the literature, however treating it remains challenging and no treatment has yet recognized 100% effective for a cure [3].

Replication of DNA viruses, such as HPV requires K + influx. Both furosemide, a loop diuretic, and digoxin, a cardiac glycoside, constrain K + influx by interrelating with cell membrane ion co-transporters (Na+/K+-ATPase and Na+-K+-2Cl-co-transporter-1). Hence, both compounds may be appreciated in the treatment of warts. This novel combination is called ionic contra-viral therapy (ICVT) [4]. Intralesional injection of combined digoxin and furosemide was observed to be effective and safe as a treatment alternative for multiple plantar warts [5].

5-Fluorouracil (5-FU) can be integrated into RNA and DNA, disrupting nucleoside metabolism and causing cell cytotoxicity and death [6]. In the treatment of warts, intralesional 5-fluorouarcil has been observed to be a greatly effective, safe and economic alternative [7].

In this randomized control study, we assessed the efficacy and safety of intralesional combined digoxin and furosemide versus intralesional 5-flurouracil in the treatment of multiple recalcitrant plantar warts.

## Patients and methods

### Patients

90 adult patients of both sexes with multiple (≥ 3) recalcitrant plantar warts were included in our study. Recalcitrant plantar warts were described as “warts of more than 2 years duration that failed to respond to at least 2 different modalities” [8]. We diagnosed warts clinically and confirmed the diagnosis by dermoscopic examination. Patients included in this work did not receive any wart management for at least 1 month before or during the study. Patients were omitted from the study if they had immunosuppression, an identified sensitivity to any of the therapeutic ingredients, abnormal serum K^+^ level, or history of cardiac diseases. Our exclusion criteria also included pregnant and lactating females as well as the presence of any local or systemic inflammation or infection.

Our study was approved by the institutional review board (IRB) of Zagazig University, Egypt (Approval code: ZU-IRB#9753). Patients gave an informed consent at the beginning of the study.

### Methods

Full history taking, general examination and complete dermatological examination were done to all patients. K + level and ECG were done before and after treatment in the patients treated with combined digoxin and furosemide. Dermoscopy, *Dermlite©DL1“Gen, USA”* attached to *Huawei nova 3i dual* camera, was used to determine warts characteristics (Papilliform surface, bleeding streaks and spots, loss of dermatoglyphics, frogspawn pattern) [9].

Patients were assigned to one of 3 groups in a random fashion, each containing 30 patients:

#### Group A

0.1 mL of combined digoxin and furosemide was received slowly into the base of each wart, with maximum 5 warts per session. The active formula preparation and dose calculation was made from digoxin *(®Lanoxin, GlaxoSmithKline company)* and furosemide *(®Lasix, Sanofi Aventis company) as recommended by *Fathy et al. [5].

#### Group B

received intralesional injection of 5- Fluorouracil *(® Utoral 500 mg/10 ml solution, Hikma Specialized Pharmaceuticals)* in full concentration till the whole lesion begins to bulge. Two ml of 5-FU per session was maximally injected.

#### Group C

received intralesional saline. Fortnightly injections were done into all studied warts using a 27- gauge insulin syringe till complete clearance or up to 5 sessions.

### Evaluation of the response

The studied warts were clinically evaluated regarding change in size (mm) as follows [10]: Complete response: wart disappearance and return of the normal dermatoglyphics; Partial response: 50–99% diminution in wart size; and no response: 0–49% diminution in wart size.

Also, response rate was classified according to decrease in number of warts as excellent: 75–100% decrease in number of warts; very good: 50–75% decrease; good: 25–50% decrease; and poor: 0–25% decrease.

Dermoscopic response of warts was evaluated 2 weeks after the last treatment session as [11]:

Score 0: neither clinical nor dermoscopic clearance; Score 1: clinical improvement without dermoscopic clearance; Score 2: clinical clearance without dermoscopic clearance; Score 3: complete clearance clinically and dermoscopically.

Pain complained by the patients was evaluated by a visual analogue scale (VAS) recording from 0 (no pain) to 10 (the maximum pain). Immediate and late adverse effects were also evaluated after each treatment session.

### Follow-up

We followed-up the patients monthly for 3 months after treatment completion to notice any wart recurrence.

### *Statistical* analysis

Data were analyzed by SPSS version 20. ANOVA (F-test), Chi-square test (X^2^), Kruskal-Wallis test (KW), and Mann-Whitney test were used. The threshold of significance was fixed at 5% level. *P*-values less than 0.05 were considered statistically significant.

## Result

90 patients (50 males and 40 females) with multiple recalcitrant plantar warts were included and completed the study. The age of the patients varied from 18 to 57 years, lesions number ranged from 3 to 18, all warts were smaller than 1 cm in diameter and the duration ranged from 5 months to 3 years. Distant warts were noted only in 16 patients in group A and in 6 patients in group B. Cryocautery and chemical cautery were the most often previously utilized therapeutic modalities. We didn’t observe any statistically significant differences in the standard features between the studied groups.

### Therapeutic response

**Fig. 1:**
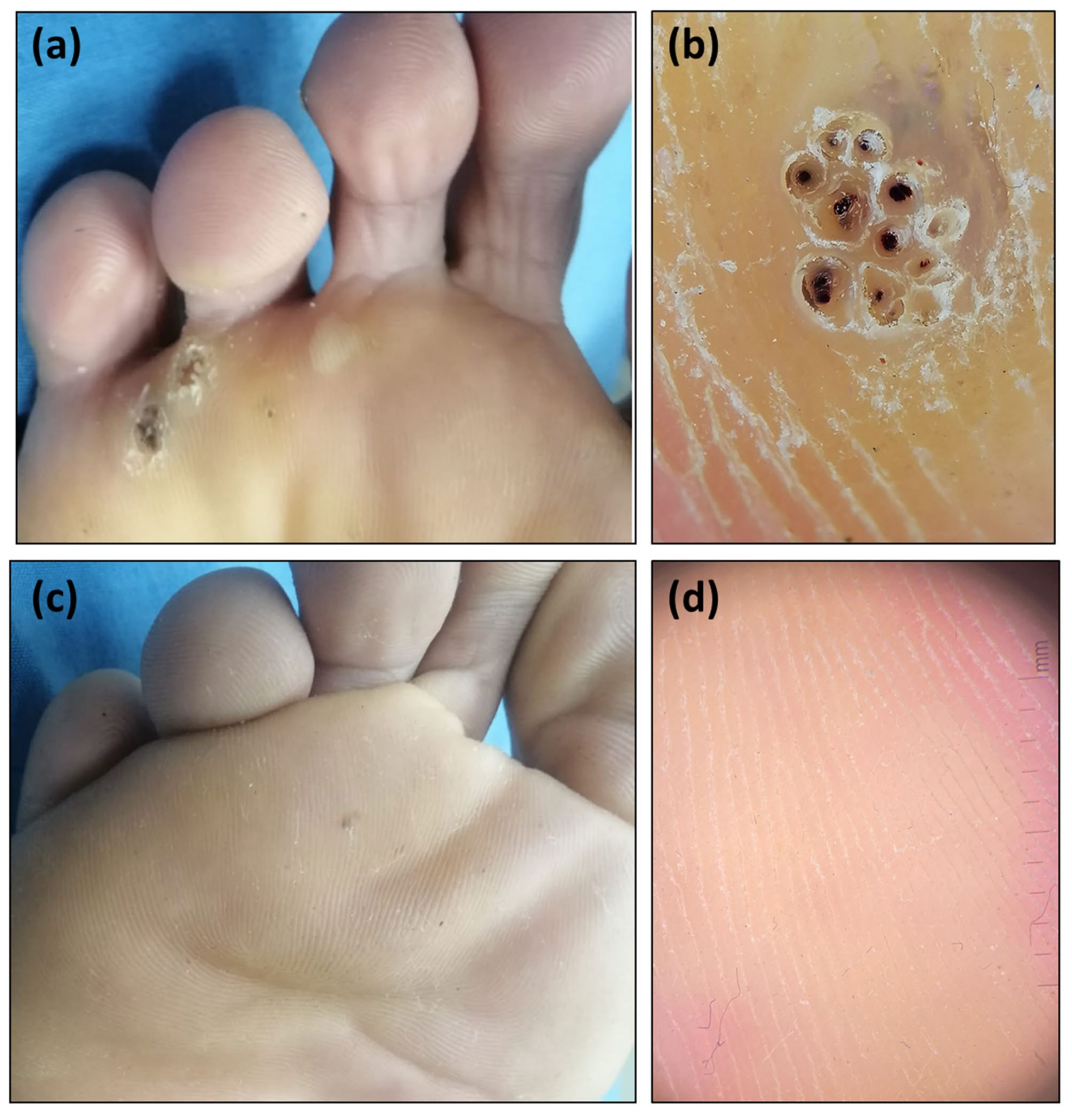
Recalcitrant multiple plantar warts on the right foot in 23 year old male patient. (**a**) Clinical image of plantar warts, (**b**) Dermoscopic image of plantar warts showing frogspawn pattern and loss of dermatoglyphics, (**c**) Complete clinical clearance of the warts after treatment with intralesional digoxin and furosemide, (**d**) Dermoscopic image showing complete clearance of warts with normal skin marking after treatment with intralesional digoxin and furosemide (score 3)

**Fig. 2:**
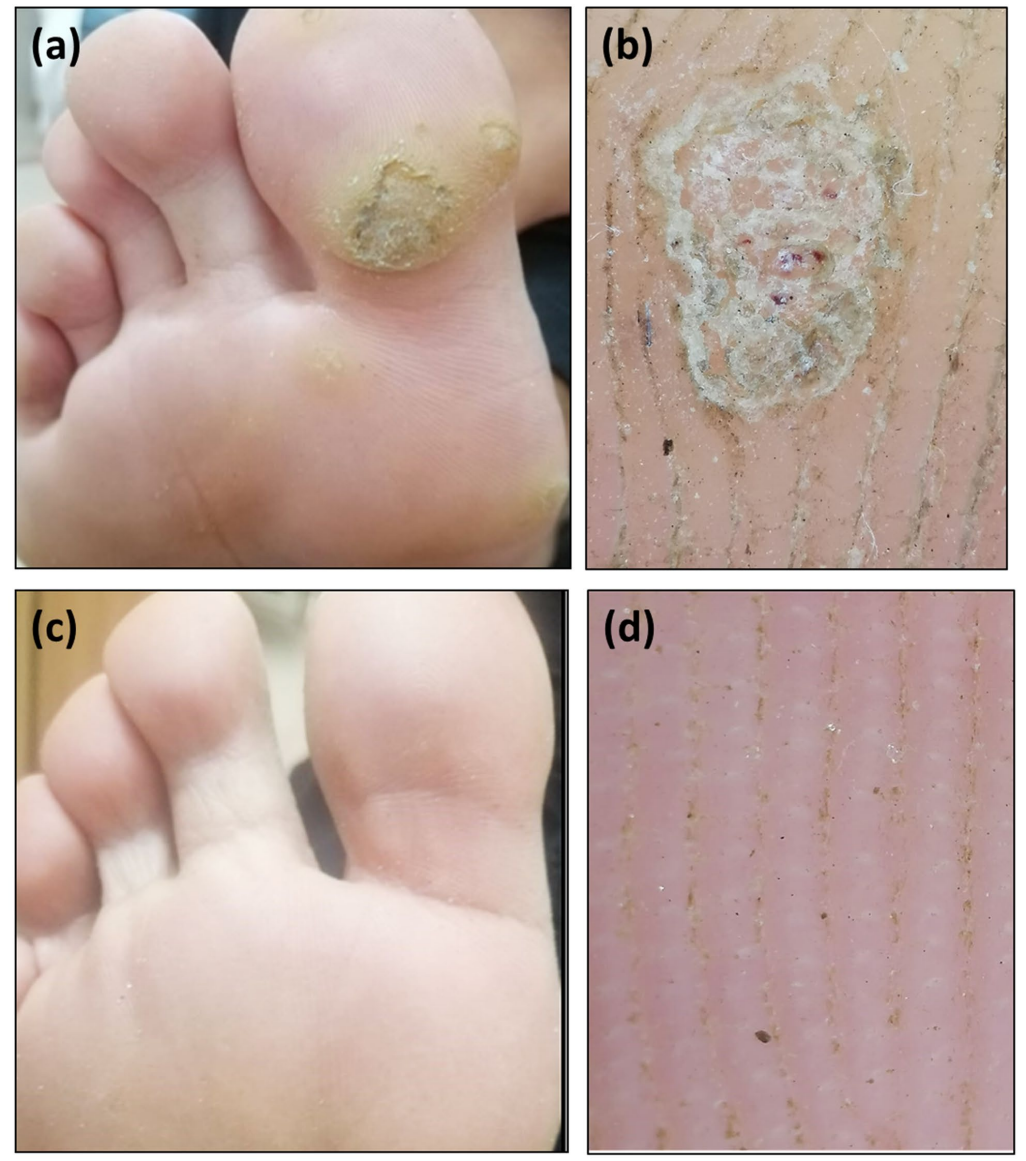
Recalcitrant multiple plantar warts on the right foot in 39 year old female patient. (**a**) Clinical image of plantar warts, (**b**) Dermoscopic image of plantar warts showing scaly yellowish structureless area with bleeding streaks and spots and loss of dermatoglyphics, (**c**) Clinical complete clearance of the warts after treatment with intralesional 5- FU, (**d**) Dermoscopic image showing complete clearance of warts with normal skin marking after treatment with intralesional 5- FU (score 3)

There were a statistically highly significant reduction in wart size and number in both digoxin & furosemide group and 5-FU group (Table 1) (Figs. 1 and 2). Among the patients achieved complete response, 6 patients (20%) achieved it after the second session and 2 patients (6.7%) after the third session in digoxin and furosemide group, while in 5-FU group, 8 patients (26.7%) showed it after the fourth session and the remaining showed this response at the end of the study.

Each treated group showed statistically highly significant decrease in wart size and number compared to saline group. However, there was no statistically significant difference between the two treated groups. About half of the patients in both treated groups achieved partial improvement in wart size and excellent response in number reduction. However, majority of the patients (93.3%) in saline group showed no response (Table 1).

**Table 1:** Response of wart size and number among the studied groups

Variable	Digoxin and furosemide group (*N* = 30)		5 - FU group (*N* = 30)		Saline group (*N* = 30)		F-test	*P*-value	LSD
**Size of wart (mm) :**									
- Before treatment:							0.141	0.986	-----
*Mean ± SD*	4.3 *±* 1.1		4.5 *±* 1.2		4.9 *±* 0.9				
*Range*	3–6		2–6		4–7				
- After treatment:									
*Mean ± SD*	1.8 *±* 1.4		2.1 *±* 1.3		4.1 *±* 1.1		11.2 (Kw)	0.000**	*P1:0.548* ***P2:0.000*** ** ***P3:0.000*** **
*Median (Range)*	1 (1–5)		2 (1–5)		5 (3–6)				
*- P*-value	0.000**		0.000**		0.689				
**Response (size):**	**N**	**%**	**N**	**%**	**N**	**%**	**χ 2**		
• *No*	6	20	6	20	28	93.3	25.9	0.000**	
• *Partial*	12	40	14	46.7	1	3.33			
• *Complete*	12	40	10	33.3	1	3.33			
**Number of wart:**									
- Before treatment:									***-----***
*Mean ± SD*	4.8 ± 0.56		6.8 *±* 4		5.1 *±* 2.4		10.1(Kw)	0.623	
*Median (Range)*	5 (3–5)		7 (4–16)		5 (3–10)				
- After treatment:									
*Mean ± SD*	2.1 *±* 2.1		3 *±* 2.8		4.9 *±* 2.3		2.9(Kw)		*P1*: 0.293 ***P2***: 0.002* ***P3***:0.033*
*Median (Range)*	2 (0–5)		2 (0–8)		4 (3–10)			0.007*	
*- P*-value	0.000**		0.000**		0.082				
**Response (number):**	**N**	**%**	**N**	**%**	**N**	**%**	**χ** ^**2**^		
• *Poor*	8	26.6	8	26.6	29	96.67			
• *Good*	6	20	8	26.7	0	0	21.9	0.001**	
• *Very good*	2	6.7	2	6.7	0	0			
• *Excellent*	14	46.7	12	40	1	3.33			

*P*1: Digoxin and furosemide group versus 5- fluorouracil group; *P*2: Digoxin and furosemide group versus saline group; *P*3: 5- fluorouracil group versus saline group; *P*#: repeated measure ANOVA; Kw: Kruskal Wallis test; 5- Fu: 5- fluorouracil; χ ^2^: chi square test; F: ANOVA test; LSD: Least significant difference

*Statistically significant difference (*P* ≤ 0.05)

**Statistically highly significant difference (*P* ≤ 0.001)

Dermoscopically, partial improvement (score 1, 2) was achieved in about half of the patients (53.4%) in digoxin and furosemide group and about two third of the patients (66.7%) in 5-FU group. Complete clearance (score 3) was achieved in 10 patients in digoxin and furosemide group and in 8 patients in 5-FU group (Figs. 1 and 2).

Regarding distant warts, 6 out of 16 patients (37.5%) in digoxin and furosemide group showed complete response. On the contrary, 4 out of 6 patients (66.7%) in 5-FU group showed partial response with no one responded completely.

### Relationship between therapeutic response and some clinical variables

No significant relationships were noted between therapeutic response in the studied groups and age, sex, number, size, duration of warts, and previous treatments. There was no relationship between serum K^+^ level in group A and therapeutic response.

### Adverse effects

Pain had been showed to be the most common side effect among the studied groups (66.6% in digoxin and furosemide group, 80% in 5-FU group and 53.3% in saline group). Haemorrhagic bullae and post-inflammatory hyperpigmentation were observed only in 5-FU group (26.7% & 13.3% of the patients, respectively).

### Recurrence

There was no relapse or new lesions appeared throughout the 3 month follow up period.

## Discussion

Although warts do not result in acute symptoms, except pain, they need treatment due to cosmetic concerns and the autoinfection risk^**2**^. Up to our knowledge, a topical formulation of combined digoxin and furosemide for the treatment of warts was studied in just 3 prior studies [4, 12, 13].

Both digoxin and furosemide inhibit K + influx, so they can affect DNA virus replication which depends on K + influx [12]. Herein, we compared the effectiveness and safety of intralesional injection of combined digoxin and furosemide and intralesional 5-fluorouarcil, an antimetabolite that suppresses cell division and causes cell cycle arrest.

In digoxin and furosemide group in our study, 40% of the patients had complete response. This response was greater than those given by **Nasr et al.** [14] who displayed complete decrease of plantar warts in 28% of patients. This may be clarified by our study was conducted on plantar warts as compared to different types of multiple warts in the mentioned study.

On the contrary, our clearance rate was poorer than that described by **Fathy et al.** [5] (50%). This may be due to their scoring system, as they considered excellent response if there was 75–100% decrease in wart size as compared to a full disappearance (100%) in our study. Furthermore, it was much lower than other studies that reported more than 90% clearance [15]. This can be elucidated by the dissimilar concentration of the combined drugs.

Comparing intralesional combined digoxin and furosemide with other intralesional therapies of warts, it was less effective (28%) than candida antigen (60%) and vitamin D3 (48%) [14].

Our results showed that 53.4% had > 50% decrease in number of warts (excellent (46.7%) and very good response (6.7%)). Our result was comparatively close to that described by **Fathy et al.** [5] who observed 50% decrease in number of warts. This result may be improved by more frequent sessions of treatment, more number of warts treated in the session, less intervals between the sessions or increasing the concentration of the active treatment.

Saline is typically utilized as the control for the treatment of warts. To increase the power of the clinical trial in our investigation, we designed the third group to receive intralesional saline as a control. There were highly statistically significant reduction in size and number comparing digoxin & furosemide and saline groups. Our results were relatively similar to other studies [5, 15].

In 5-fluorouracil group, 80% of the patients showed complete and partial clinical response (33.3% and 46.7%, respectively). This response was comparatively close to that observed by other studies [7, 16].

Our result was poorer than that noted by other studies [17, 18]. More number of sessions or the assessment of response in a longer follow-up period (12 weeks) could be the explanation.

There were highly statistically significant difference between 5FU and saline group as regard reduction in number and size. Our results were relatively parallel to that described by Iscimen et al. [19].

Comparing Intralesional 5-FU injection with others, it was less effective than intralesional bleomycin and candida antigen in the treatment of plane warts [20]. Howevere, it was more effective than intralesional Bacille-Calmette-Guérin in handling different types of warts [21].

Among the patients achieved complete response in 5-FU group (33.3%), only 26.7% showed clearance after the fourth session and the remaining showed this response at the end of the study. Howevere, **Srivastava et al.** [18] observed full clearance in 95.4% of warts after one or two injections. This difference can be attributed to different type of warts (palmoplantar in their study and plantar in ours).

Dermoscopically, complete clearance achieved in 10 patients in digoxin and furosemide group (clinically achieved in 12 patients) and in 8 patients in 5-FU group (clinically achieved in 10 patients). Therefore the dermoscope allowed accurate grading and deciding the end point of management rather than the preceding approaches that were dependent only on clinical inspection.

Up to our awareness, this is the first study to compare the efficacy and safety of intralesional injection of combined digoxin and furosemide versus intralesional 5-fluorouarcil in the treatment of multiple plantar warts. There was no statistically significant difference between both groups.

Complete clearance of untreated distant warts in combined digoxin and furosemide group (37.5%) in our study added to the probable benefits of this medication in the treatment of multiple plantar warts. It could be explained by the assumption of distant antiviral influence or due to activation of the immune system. The immune response is known to be simulated by cardiac glycosides including digoxin [22]. In contrast, the deficiency of complete response of the distant warts in 5-fluorouracil group may mirror the purely localized, cytotoxic effect on HPV-laden cells.

In agree with our study, pain was stated as a persistent side effect for 5-fluorouracil injection [16, 18, 19]. Haemorrhagic bullae and post-inflammatory hyperpigmentation were unique side effects in 5-FU group as reported also in other works [16, 18, 21]. Bullae formation is believed to be a therapeutic effect rather than a side effect as the degree of complete clearance was more rapidly when the bulla is formed.

Our findings were in accordance with other studies [5, 12] regarding cardiac safety and tolerability (as no systemic affection by ECG and serum K^+^).

In conclusion, Intralesional injection of 5-fluorouarcil and combined digoxin and furosemide are nearly equivalent in efficacy and safety for plantar wart treatment. We recommend utilizing dermoscopy in the treatment of warts to decide the end point of treatment consequently reducing the chance of recurrences.

Further studies are recommended on large number of patients with further work on evaluation of more number of treatment sessions and comparing intralesional with topical mode of combined digoxin and furosemide administration. Further large studies are recommended with long follow-up to assess possible recurrences and confirm long-term efficacy.

## Data Availability

No datasets were generated or analysed during the current study.

## Abbreviations

5-FU: 5- Fluorouracil
HPV: Human papilloma virus
ICVT: Ionic contra-viral therapy
